# Prediction Equations to Estimate Resting Metabolic Rate in Healthy, Community-Dwelling Chinese Older Adults

**DOI:** 10.3390/nu18020344

**Published:** 2026-01-21

**Authors:** Zhenghua Cai, Bochao You, Shuyun Yu, Yi Fan, Haili Tian, Barbara E. Ainsworth, Peijie Chen

**Affiliations:** 1 School of Exercise and Health, Shanghai University of Sport, Shanghai 200438, China; chasecome@163.com (Z.C.); 2421517013@sus.edu.cn (B.Y.); 2421517015@sus.edu.cn (S.Y.); 2521517004@sus.edu.cn (Y.F.); tianhaili123@163.com (H.T.); barbara.ainsworth@asu.edu (B.E.A.); 2 College of Health Solutions, Arizona State University, Phoenix, AZ 85003, USA

**Keywords:** resting energy expenditure, elderly Chinese population, prediction models, calorimetric measurement

## Abstract

**Background**: China’s rapidly aging population demonstrates the importance of conducting an accurate resting metabolic rate (RMR, kcal/day) assessment to mitigate geriatric nutritional imbalances—amid concurrent undernutrition (e.g., ~1/3 with protein insufficiency) and overnutrition (e.g., high obesity and type 2 diabetes rates). While RMR prediction equations exist for other populations, none are specific to Chinese older adults. This study aimed to develop and validate population-specific RMR prediction equations for community-dwelling Chinese older adults. **Methods**: A total of 189 healthy participants (Aged 69.5 ± 6.3, range: 60–94 years; BMI: 24.0 ± 3.1 kg/m^2^) were recruited from the Shanghai, China, community. RMR was measured via indirect calorimetry, and body composition via dual-energy X-ray absorptiometry. **Results**: Two novel prediction equations were derived: Cai1 (fat-free mass [FFM] + age): RMR = 1393.019 − (11.112 × age) + (11.963 × FFM); R^2^ = 0.572, and Cai2 (sex + age + weight [WT]): RMR = 1537.513 + (91.038 × sex) − (11.515 × age) + (5.436 × WT); R^2^ = 0.528. Both novel prediction equations achieved 82.5% adequacy (predicted RMR within 90–110% of measured values), minimal systematic bias (%) (−0.72% and −1.08%) and strong positive correlations with measured RMR (r = 0.792 and 0.773, both *p* < 0.001). Bland–Altman analysis confirmed no systematic bias. In contrast, 11 widely used published prediction equations (e.g., Harris–Benedict, Mifflin–St. Jeor) exhibited significant overestimation (systematic bias +8.39% to +38.03%). **Conclusions**: The novel population-specific RMR equations outperform published ones, providing a clinically reliable tool for individualized energy prescription in nutritional interventions to support healthy aging in Chinese older adults.

## 1. Introduction

China’s accelerated population aging has placed health challenges on older adults, particularly nutritional imbalances, at the forefront of public health concerns [1]. With individuals aged 60 and older exceeding 290 million and accounting for over 21% of the total Chinese population [2], this demographic trend underscores an urgent need to address age-related nutritional issues [3]. Epidemiological studies reveal a “dual burden” of malnutrition prevalent in this population: undernutrition, characterized by significant protein insufficiency affecting approximately one-third of Chinese older adults, elevates the risk of sarcopenia and immune dysfunction; and overnutrition, contributing to high rates of overweight, obesity, type 2 diabetes, and hypertension [4]. Age-associated physiological decline, including diminished digestive function and reduced physical activity, exacerbates these nutritional imbalances [5]. Consequently, a precise assessment of energy requirements is essential for developing targeted nutritional interventions to mitigate these risks and promote healthy aging [4].

Total Daily Energy Expenditure (TDEE) in kilocalories per day (herein referred to as kcal) is primarily determined by three components: the thermic effect of food, energy expended through physical activity, and the resting metabolic rate (RMR). RMR, defined as the energy required to sustain fundamental physiological functions while in a state of complete rest [6,7], accounts for the largest share of TDEE, ranging from 60% to 75% of TDEE in most adults [8,9]. A well-established characteristic of RMR is its progressive decline with age, decreasing at an average rate of 1% to 2% per decade from early adulthood onward [10]. As the most significant component of TDEE that declines with age, RMR serves as the core baseline for estimating older adults’ energy requirements. By integrating RMR with the thermic effect of food and activity-related energy expenditure, precise daily calorie intake can be derived. This integration will directly support targeted nutritional interventions to address the dual burden of undernutrition and overnutrition.

Indirect calorimetry (IC) is established as the reference standard technique for determining RMR [11]. IC quantifies energy expenditure by measuring oxygen consumption (VO_2_) and carbon dioxide production (VCO_2_), based on the principle that substrate oxidation is linked to respiratory gas exchange. The IC method has been extensively validated across diverse populations, including healthy individuals, athletes, and clinical patients. It is regarded as the most accurate approach for quantifying RMR in both research and clinical practice [12].

Despite its strong validity, the routine application of IC is limited by practical issues. These include the high costs of specialized laboratory equipment (e.g., metabolic carts and precise gas analyzers) and the need for trained personnel to conduct calibrations and ensure measurements are taken under standardized resting conditions [13]. While portable IC devices (e.g., COSMED K5) have been developed as more accessible alternatives to laboratory-based measurements, laboratory and portable IC measurement methods remain less feasible for widespread use than prediction equations, especially in routine clinical or field settings.

Consequently, prediction equations developed from variables such as sex, age, height, and weight are widely adopted as the primary method for assessing RMR in individuals. The most common prediction equations used include the Harris–Benedict [14], FAO/WHO/UNU [15], Schofield [16], Owen [17] and Mifflin–St. Jeor [18], with the latter recommended by the American Dietetic Association [19]. Other RMR prediction equations have been developed that incorporate body composition as a predictive variable (e.g., Cunningham [20]). Others are tailored for older adult populations (e.g., Fredrix [21], with participants’ mean age = 65 ± 8 years) and for distinct populations (e.g., Liu [22], Wang [23], and Xue [24] for Chinese populations).

While published RMR prediction equations have been extensively applied across diverse adult populations, most are not suitable for estimating RMR in Chinese older adults. For example, a critical limitation of the WHO equation was its dependence on the Schofield database. Specifically, 3388 of 7173 participants were Italian, with a paucity of participants from tropical regions, leading to disproportionate representation of Western European populations. Beyond ethnic differences, older adult populations are unique in that they exhibit a decline in RMR due to increased fat mass and reduced muscle mass [9]. Previous studies have confirmed that the Chinese older adult population residing in Mainland China has an even lower RMR than other older adult populations living elsewhere [25]. Furthermore, recent studies investigating RMR in older adults from other Asian countries have consistently demonstrated that RMR values predicted by published equations derived from Western populations significantly overestimate the actual RMR observed in this demographic subgroup [26]. Consequently, the use of existing RMR prediction equations in Chinese older adults introduces significant prediction errors, preventing accurate estimation of the kcal energy intake needed to address undernutrition and overnutrition. Accordingly, more precise RMR prediction equations are required to quantify RMR in Chinese older adults.

This study was conducted to develop a population-specific RMR prediction equation for community-dwelling Chinese older adults residing in Mainland China. The study’s purpose was threefold: first, to measure the RMR of Chinese older adults; second, to identify the errors associated with applying published RMR prediction equations in Chinese older adults; and third, to develop a specialized RMR prediction equation for use in the relatively healthy Chinese older population residing in Shanghai, China. This work aims to address the upcoming challenges of malnutrition and overnutrition posed by China’s aging society.

## 2. Materials and Methods

### 2.1. Study Design and Participants

This study used an analytical cross-sectional design. One hundred eighty-nine Chinese older adults residing in various communities in Shanghai, China (aged 69.5 ± 6.3, range: 60–94 years), volunteered to participate in the study. All participants met the following inclusion criteria: aged ≥60, general good health, and the absence of hypermetabolic diseases (e.g., cancer, diabetes, anemia, or thyroid disease), and a stable body weight for the last 6 months [27,28,29]. Participants were excluded if they had moderate hypertension [30], defined as resting systolic blood pressure > 159 mmHg or diastolic blood pressure > 99 mmHg and were taking medication or a hormone that modifies RMR.

Before the study, participants provided informed consent in accordance with the ethical standards of Shanghai University of Sport. The study procedures were conducted in accordance with the ethical principles outlined in the Declaration of Helsinki and the Council of International Organizations of Medical Sciences (Ethical number: 102772024RT007, Approval date: 24 May 2024).

### 2.2. Measurements

Inclusion and exclusion criteria were measured with a questionnaire developed for this study. Systolic and diastolic blood pressures were measured with a UDEX-i2 monitor (Canon Medtech Supply Corporation, Kawasaki-shi, Kanagawa, Japan) following standardized protocols.

Before all physical measurements, participants removed shoes and heavy clothing, abstained from caffeine, smoking, and moderate-to-vigorous physical activity for 24 h, and maintained an overnight fast for at least 12 h before the RMR measurement. All participants were asked to rest for 15 to 20 min prior to the measurement. Physical activity restrictions included intentional physical activity (e.g., walking or jogging for exercise, bicycling for transportation or pleasure, participation in exercise classes, and use of gymnasium equipment). All measurements were conducted between 07:00 and 09:00 to minimize the impact of diurnal variations on outcome measures.

Demographic characteristics and anthropometric parameters were collected before the RMR measurement to detect participants’ variables for prediction equations. Age was measured in years. Body weight (kg) and height (cm) were measured using calibrated laboratory scales, and body mass index (BMI) was computed as weight in kg divided by height in meters squared. Body composition, including fat mass (kg), fat mass percent (%), and fat-free mass (kg), was quantified using a GE Lunar iDXA scanner (GE Healthcare, Madison, WI, USA).

RMR was assessed via IC using a Quark Cardiopulmonary Exercise Test system (COSMED, Rome, Italy). The system’s flow meter and gas analyzer were calibrated before each test. Measurements were performed indoors under controlled environmental conditions (temperature: 24–26 °C; relative humidity: 45–70%; 750–770 mmHg) with dimmed lighting to minimize external stimuli. Participants reclined supine on a laboratory bed for 60 min, remaining quiet and awake throughout the measurement period. A standard pillow was used to maintain head elevation, ensuring unobstructed airflow through the canopy placed over the participant’s head. Metabolic parameters—oxygen consumption (VO_2_), carbon dioxide production (VCO_2_), and respiratory quotient (RQ = VCO_2_/VO_2_) were recorded following attainment of a stable period, defined as no significant fluctuations in VO_2_, VCO_2_, or RQ. Test validity was confirmed by RQ values within the physiological range (0.7–1.0) [12]. VO_2_ was averaged from the 5th minute of rest to 2 min before the end of the measurement. Steady state was defined as the first 5 min period with a coefficient of variation (CV) ≤ 10% for both VO_2_ and VCO_2_ [31], VO_2_ and VCO_2_ remaining constant, Minute Ventilation (VE) stabilizing within ±5–10% and heart rate (HR) stabilizing within ±5%. The %CV is the ratio of the standard deviation (SD) to the mean ($\bar{x}$) and is calculated as [(SD/$\bar{x}$) × 100]. Aberrant values (e.g., abrupt spikes or drops in VO_2_) were excluded to ensure data consistency. RMR was calculated using the abbreviated Weir equation: 1.440 × ((3.9 × VO_2_) + (1.1 × VCO_2_)) [32], with VO_2_ and VCO_2_ converted from mL/min to L/min before calculation.

To evaluate internal validity and control for Type I error, the total sample was stratified by sex and age, then randomly split into a development subsample (70%, n = 132; 51 males, mean ± SD age = 70.9 ± 5.8 years; 81 females, mean ± SD age = 68.2 ± 6.0 years) and a validation subsample (30%, n = 57: 22 males, mean ± SD age = 70.5 ± 6.8 years; 35 females, mean ± SD age = 69.6 ± 6.6 years). Stratified random sampling ensured balanced distribution of sex and age between the two subsamples.

Cross-validation of two novel and 11 published prediction equations was performed in the validation total sample and by sex. Sex-specific analyses addressed potential sex-related heterogeneity. Sex-specific published prediction equations [14,15,16,17,18] were applied to male and female subsamples separately; combined prediction equations [20,21,22,23,24] were applied to males and females equally when prediction equations were not sex specific.

### 2.3. Published RMR Prediction Equations

Eleven published RMR prediction equations are presented in Table 1. Five equations [14,15,16,17,18] are sex-specific; others [20,21,22,23,24] present only one equation for males and females combined. Three equations [14,18,22] include age, height, and body weight as predictive variables, two [21,24] include body weight and age, three [15,16,17] include only body weight, one [24] includes fat-free mass and age, and two [20,23] include only FFM as a predictive variable.

### 2.4. Statistical Analysis

Quantitative variables were described using measures of central tendency and dispersion and are presented as means ± standard deviations ($\bar{x}$ ± sd). Data distribution normality was assessed using the Shapiro–Wilk test. Stepwise linear regression and quadratic regression models (adding age^2^) were explored to determine the influence of the participant’s age range (60 to 94) on the prediction of RMR. Two novel RMR prediction equations (Cai equations 1 (Cai1) and 2 (Cai2)) were developed in the study development subsample using stepwise regression analysis, with automatic checks for the Variance Inflation Factor (VIF) and the Durbin–Watson (D–W) test to detect multi-collinearity of the predictive variables and autocorrelation of the residuals, respectively. Descriptive and anthropometric variables were used as predictors. For Cai1, predictive variables included those that did and did not require DXA screening (age, sex, height, weight, BMI, and FFM). For Cai2, predictive variables included those that did not require DXA screening (age, sex, height, weight, and BMI). The dependent variable was the measured RMR. Examination of the model coefficients (β (95% CI) and SEs) and *p*-values for the variables used in the RMR prediction equations was conducted. Differences between the two novel RMR equations were determined using a paired *t*-test. Three approaches were used to directly address uncertainty in the novel prediction equations. First, a 10-fold cross-validation on the total sample (N = 189) was used to compare the regression equation coefficients in the development sample (n = 132) with the total sample. Second, intraclass correlation coefficients (ICC) were used to evaluate agreement between the novel prediction equations and the measured RMR. Koo’s [33] classification is used to rate the strength of the ICC (0.91–1.00 = excellent agreement, 0.75 to 0.90 = good agreement, 0.50 to 0.74 = moderate agreement, and <0.21 = poor agreement). Third, an age-stratified sensitivity analysis with internal bootstrap validation (1000 resamples) of the novel prediction equations was used to assess the stability of the regression coefficients.

Differences between the 13 RMR prediction equations (11 published and 2 novels) and the measured RMR were compared in the validation subsample using the Repeated Measures Analysis of Variance (RM-ANOVA). Dunn’s post hoc comparisons (t statistic), with a Bonferroni correction for multiple comparisons, were performed when a significant RM-ANOVA F statistic was found. The mean difference ($\bar{x}$_d) between the predicted and measured mean RMRs was calculated by subtracting the predicted RMR from the measured RMR [34]. A positive difference indicated that the predicted RMR kcal was higher than the measured value.

Box-and-whisker plots were generated to visualize the distributions, spreads, and skewness of the predicted and measured RMRs. The plots show the kcal values for outliers, the minimum and maximum, median, and first and third quartiles.

The Pearson product–moment correlation coefficient (r) examined the strength and direction of the linear relationship between the predicted and measured RMRs. Chan’s classification was used to rate the strength of the correlation (≥0.8 = very strong, 0.6 to <0.8 = moderately strong, 0.3 to <0.6 = fair, <0.3 = poor) [35].

Bland–Altman analyses assessed the precision of the predicted and the measured RMR values. Analyses included the bias (systematic differences) expressed as the $\bar{x}$_d ± SD (predicted–measured kcal), the 95% upper and lower limits of agreement (LoA) showing where 95% of the differences in kcal occurred, the 95% confidence interval (95% CI) of agreement estimating the size of possible sampling error, and systematic bias % ((predicted RMR − measured RMR/measured RMR) × 100). Bland–Altman plots are displayed for the Cai1 and Cai2 prediction equations only. The plots were generated using MedCalc software version 23.2.7 (MedCalc Software bvba, Ostend, Belgium).

The percent adequacy of the prediction equations was calculated to identify overestimation and underestimation of the RMR prediction equation compared to the measured RMR. Percent adequacy was calculated as: (RMR prediction − RMR measured) × 100 [19]. The percent adequacy criteria were as follows: accurate prediction: a percent difference within ±10% (i.e., 90–110% of the measured value); underestimation: a difference of <−10% (i.e., <90% of the measured value); and overestimation: a difference of >+10% (i.e., >110% of the measured value). Prediction accuracy was defined as the percentage of participants whose predicted RMR was within ±10% of the measured RMR. All other prediction accuracies were treated as errors and reported as the percentage of participants whose RMR was underestimated or overestimated. *p* < 0.05 was considered statistically significant. All statistical analyses were performed using IBM SPSS Statistics 28.0 (IBM Corp., Armonk, NY, USA).

## 3. Results

### 3.1. Subject Characteristics

All variables were normally distributed. Participants included 189 adults (73 males, 116 females) aged 60 to 94. Table 2 shows the descriptive, anthropometric, and cardiovascular variables of the study participants. Age, body weight, height, fat-free mass, measured RMR, and systolic and diastolic blood pressure values were higher in males than in females. Fat mass and fat mass percent were higher in females than in males. There was little difference in BMI between males and females.

### 3.2. RMR Prediction Equations from the Development Subsample

Table 3 presents two stepwise regression RMR prediction equations arising from the stepwise linear regression model that were generated from the development subsample (n = 132). The resulting Cai1 equation included age and FFM as predictive variables. The Cai2 equation included age, sex, and body mass as predictive variables.

The proportion of variance in the measured RMR explained by the prediction equations (R^2^) was similar in both equations (Cai1, R^2^ = 0.572; Cai2, R^2^ = 0.528). FFM was the strongest predictor of the measured RMR (β_RMR_ = 0.632) among the variables in each prediction equation. VIFs for all variables in the equations were <5, indicating no multicollinearity, and D–W was 1.631, which is close to 2, suggesting no autocorrelation among the residual terms. The root mean squared error (RMSE) shows how far the predicted RMR values are from the measured values. The RMSEs are 99.238 kcal for the Cai1 equation and 104.189 kcal for the Cai2 equation. The paired *t*-test showed no significant difference in predicted RMR between Cai1 (RMR = 1117.0 ± 125.6 kcal) and Cai2 (RMR = 1121.0 ± 112.7 kcal) (t = −0.984, *df* = 56, *p* = 0.329). The regression coefficient of the quadratic model including age^2^ for Cai1 was 0.160 (t = 1.332, *p* = 0.185 > 0.05) and for Cai2 was 0.132 (t = 1.044, *p* = 0.298 > 0.05), interpreted as age^2^ having no statistically significant effect on equations Cai1 and Cai2.

Table 4 presents the β (95% CI), SEs, and *p*-values for the variables used in the RMR prediction equations. All coefficients were statistically significant at *p* < 0.001. None of the β (95% CI) included zero, and the SEs were low, except for sex in the Cai2 equation. The coefficient sizes, except for sex, indicate that the sample mean is an accurate estimate of the measured mean values. The wide β 95% CI and large SE for the predictive variable sex reflect variability in physical differences between males and females, which lowers correlations for females in RMR prediction equations.

### 3.3. Assessment of Uncertainty and Dispersion in the Study Samples

A 10-fold cross-validation on the total sample (N = 189) confirmed the certainty of the regression equation from the development sample (n = 132). The regression coefficients were similar between the models, confirming that the Cai1 and Cai2 prediction equations were stable and free of bias. The results are presented in Supplementary Table S1.

ICCs were calculated to determine absolute agreement between the predicted and measured RMR in the validation sample. The single-measure ICC (A,1) was 0.793 (95% CI: 0.672–0.873) for Cai1 and 0.765 (95% CI: 0.632–0.855) for Cai2, and the average measure ICC (A, K) was 0.884 (95% CI: 0.804–0.932) for Cai1 and 0.867 (0.774–0.922) for Cai2. All ICCs were rated as having good agreement (Supplementary Table S2).

Age-stratified sensitivity analysis with internal bootstrap validation (1000 resamples) in the Cai1 and Cai2 prediction equations revealed unusually high values for Cai1 and Cai2 equations (slope *B* (95% CI) = 1.541 (1.355–1.748) and 1.654 (0.784–2.524) and Intercept (95% CI) = 382.769 (−541.525–194.784) for Cai1 and 513.158 (−663.496–437.251) for Cai2 in participants aged ≥80. These values indicate that the prediction equations are not valid for use in Chinese adults aged ≥80 (Supplementary Table S3).

**Figure 1:**
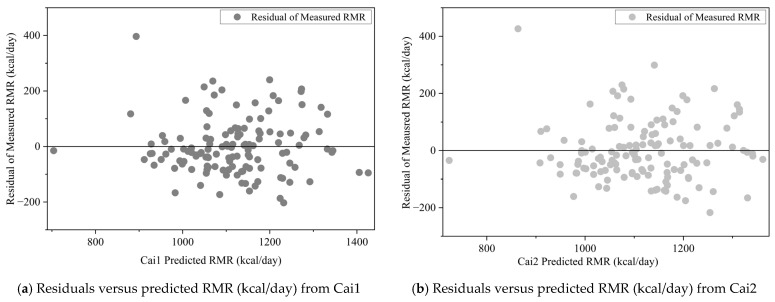
Residuals versus predicted RMR (kcal/day) equations plots in the development subsample. (**a**) Residuals versus predicted RMR from Cai1 equation and (**b**) residuals versus predicted RMR from Cai2 equation. Dark gray dot: regular residuals of RMR compared to predicted RMR from Cai1 equation; light gray dot: regular residuals of RMR compared to predicted RMR from Cai2 equation.

Figure 1 presents the residual plots (residuals versus predicted RMR for the Cai1 and Cai2 equations, where residuals are the differences between measured RMR and the predicted RMR from the Cai1 and Cai2 equations. A positive residual indicates an underestimation by the prediction model, and a negative residual indicates an overestimation.

In both plots, residuals are randomly dispersed around the horizontal reference line (residual = 0) across the entire spectrum of predicted RMR values, with no discernible linear/curvilinear trends or heteroscedastic patterns (e.g., funnel-shaped dispersion). This random distribution indicates that the new prediction equations do not introduce systematic bias in RMR estimation for Chinese older adults. Additionally, the consistent residual variance across all predicted values confirms that the homoscedasticity assumption (a critical prerequisite for the validity of linear regression-based equation derivation) is reasonably satisfied for these populations. A small number of marginal outlying residuals (e.g., the residual ~400 kcal in Figure 1b) correspond to extreme individual measurements; the outlying data points did not distort the overall distribution pattern. Collectively, these residual plots support the reliability of the new prediction equations in RMR estimation among Chinese older adults.

### 3.4. Comparison of RMR Prediction Equations with the Measured RMR

Table 5 presents the $\bar{x}$_ds and post hoc t-value for the published and novel RMR prediction equations relative to the measured RMR. RM-ANOVA confirmed a highly significant main effect of RMR prediction equations (F = 188.058; Greenhouse-Geisser corrected *df*: 2.205 [effect] and 123.463 [error]; *p* < 0.001; partial η^2^ = 0.771), indicating substantial differences in the predicted and measured RMR values.

Post hoc pairwise comparisons showed that the mean kcal values from the 11 published equations significantly overestimated RMR (all *p* < 0.001). The $\bar{x}$_ds between the predicted RMR and the measured RMR kcal values ranged from +97.1 kcal to +520.8 kcal, with the Fredrix equation [21] showing the most pronounced difference (+520.8 kcal). The Cai1 and Cai2 equations showed no significant deviations from the measured RMR, with $\bar{x}$_ds of +8.0 kcal (t = −0.53, *p* = 1.000) and +12.0 kcal (t = −0.80, *p* = 1.000), respectively.

In males, all 11 published prediction equations significantly overestimated RMR (all *p* < 0.001). The $\bar{x}$_ds values ranged from +201.9 kcal to +520.3 kcal, with Xue1 [24] performing the most overestimation. In contrast, in females, nine published prediction equations significantly overestimated RMR (all *p* < 0.001). $\bar{x}$_ds values ranged from +102.6 kcal to +610.9 kcal, with the Fredrix equation [21] showing the most overestimation (+610.9 kcal). Cai1, Cai2, Mifflin [18] and Wang [23] RMR prediction equations showed no significant differences compared to the measured RMR, with $\bar{x}$_ds of +1.5 kcal (t = 0.12, *p* = 1.000), +5.9 kcal (t = 0.46, *p* = 1.000), +35.8 kcal (t = 2.76, *p* = 0.249) and +31.3 kcal (t = 2.4, *p* = 0.470), respectively.

### 3.5. Box-And-Whisker Plots to Visualize RMR Values

**Figure 2:**
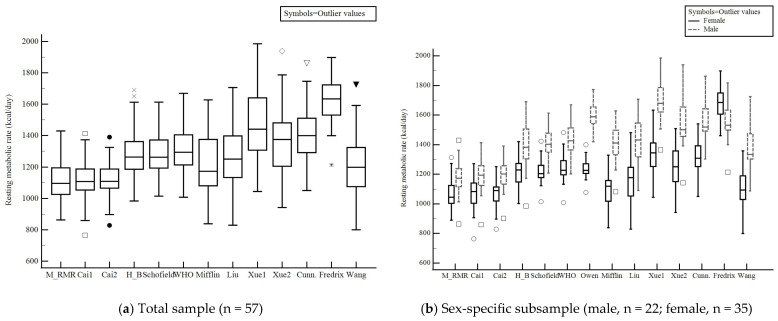
Box-and-whisker plot shows the distribution, spread, and skewness of the RMR (kcal/day) obtained from prediction equations (11 published equations and 2 Cai equations) and the measured RMR in the validation subsample. (**a**) shows total validation sample and (**b**) shows the sex-specific validation subsample of 22 males and 35 females. Solid line: female; gray dotted line: male; Unique special symbols represents different outlier cases; M_RMR: Measured RMR; Cai1: Cai equation 1; Cai2: Cai equation 2; H–B: Harris–Benedict [14]; Mifflin: Mifflin–St. Jeor [18]; Cunn: Cunningham [20].

Figure 2a presents a box-and-whisker plot of the distribution, spread, and skewness of predicted and measured RMRs. The box-and-whisker plots were similar between the Cai1 and Cai2 predicted RMR and the measured RMR. However, the other 11 prediction equations were skewed, with a relatively large spread and distribution, leading to overestimation of the measured RMR.

Figure 2b compares sex-specific RMRs with measured RMRs. In the male subsample, all prediction equations were overestimated, except Cai1 and Cai2. In females, Cai1, Cai2, Mifflin [18], and Wang [23] yielded predicted RMRs similar to the measured RMRs, whereas the other nine equations were skewed, leading to overestimation of the measured RMRs.

### 3.6. Correlations Among the Predicted and Measured RMR Values

Table 6 presents Pearson product–moment correlations among the RMR prediction equations and the measured RMR for the total sample and by sex. All comparisons with the measured RMR were statistically significant in males and in the total sample (*p* < 0.01). The correlations ranged from moderately strong (r = 0.6 to <0.8) to very strong (r ≥ 0.8) in men, and from fair (r = 0.3 to <0.6) to moderately strong in the total sample. Fewer comparisons reached statistical significance in females (*p* < 0.05) and were rated from poor to moderately strong (r = 0.3 to <0.6). The Cai1 and Cai2 equations were rated as very strong in males (r ≥ 0.8) and moderately strong in females and in the total sample (r = 0.6 to <0.8).

### 3.7. Bland–Altman Analyses of the Predicted and Measured RMR Values

Table 7 presents a Bland–Altman analysis ($\bar{x}$_d, 95% LoA, and the 95% CI of the $\bar{x}$_d) to assess the concordance between the 13 prediction equations and the measured RMR in the total and sex-specific subsamples. In the total sample, the Cai1 and Cai2 novel equations showed a low $\bar{x}$_d (≤+12 kcal), 95% LoA (from ~−150 to +175 kcal), and 95% CIs of the $\bar{x}$_d (from ~−14 to +25 kcal), confirming no systematic bias in estimating the measured RMR. In contrast, all 11 published equations showed $\bar{x}$_d > +100 kcal, wide and positive LoAs, and 95% CIs of the $\bar{x}$_d, indicating a systematic bias toward overestimating the measured RMR.

Among males, the Cai1 and Cai2 equations showed a slight $\bar{x}$_d (≤+20 kcal, with narrow 95% LoA and 95% CI of the $\bar{x}$_d ranging from negative to positive kcal values). All 11 published equations showed a consistent systematic bias toward overestimating the measured RMR, as reflected in the wide $\bar{x}$_ds, the wide ranges of positive lower and upper limits of the LoA, and the 95% CI of the $\bar{x}$_d values. The Xue1 [24], Owen [17], and Cunningham [20] equations showed the highest overestimation in predicting RMR.

Among females, the Cai1 and Cai2 equations showed a $\bar{x}$_d of less than +6 kcal, and the LoA and 95% CI of the $\bar{x}$_d were evenly distributed with values ranging from negative to positive kcal. The 11 published equations showed heterogeneous performance, unlike the consistent overestimation observed in males. Based on $\bar{x}$_d, the Fredrix [21] equation showed the most considerable overestimation in predicted RMR, followed by the Xue1 [24] and Cunningham [20] equations. While the Mifflin–St. Jeor [18] and the Wang [23] equations were not biased, their $\bar{x}$_d and LoAs led to high inter-individual variability in the predicted RMR. The remaining nine equations had wide ranges of positive lower and upper limits of the LoA and 95% CI of the $\bar{x}$_d values, indicating substantial overestimation of the measured RMR.

### 3.8. Systematic Bias (%) Derived from a Bland–Altman Analysis

**Figure 3:**
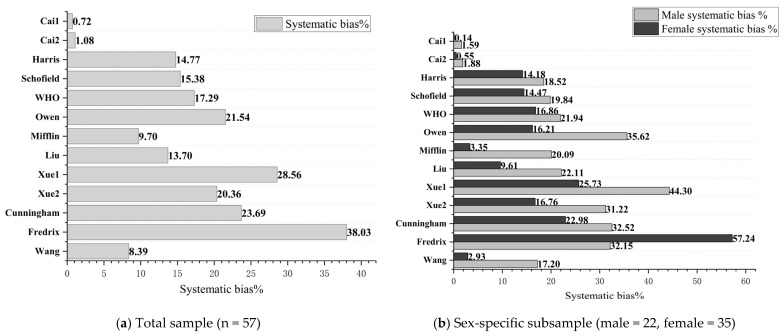
Systematic bias (%) for 13 RMR (kcal/day) prediction equations. (**a**) Total validation subsample (n = 57) and (**b**) by sex (males = 22; females = 35); Harris: Harris–Benedict [14], Mifflin: Mifflin–St. Jeor [18].

Figure 3a shows the systematic bias (%) from a Bland–Altman analysis assessing the concordance between the 13 prediction equations and the measured RMR, in the total subsample (a) and stratified by sex (b). As shown in Figure 3a, the Cai1 and Cai2 equations maintained a consistent systematic bias of ≤+1.08%. All 11 published equations showed consistent bias, ranging from +8.39% (Wang [23]) to +38.03% (Fredrix [21]).

In Figure 3b, the systematic bias for males and females was low in the Cai1 and Cai2 equations (males ≤ +1.90%; females ≤ +0.60%). Males and females in the 11 published equations showed significant systematic bias, ranging from +17.20% (Wang [23]) to +44.30% (Xue1 [24]) in males and from +2.93% (Wang [23]) to +57.24% (Fredrix [21]) in females.

### 3.9. Bland–Altman Plots of Agreement for the Cai1 and Cai2 Predicted RMR Equations

**Figure 4:**
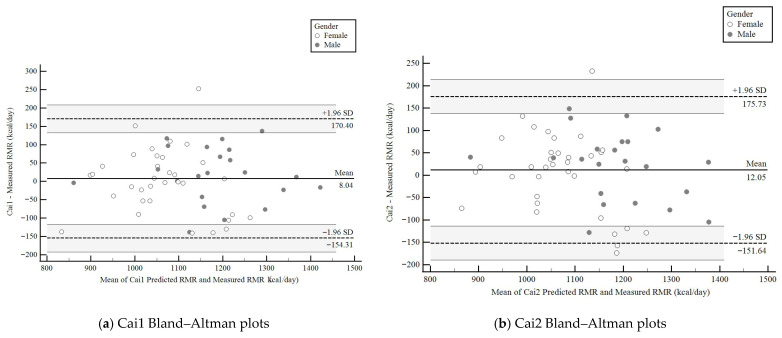
Bland–Altman plots of agreement between Cai1 (**a**) and Cai2 (**b**) predicted RMR (kcal/day) and the measured RMR in the validation subsample (n = 57; males = 22; females = 35). Solid gray circle: male (n = 22); hollow circle: female (n = 35).

Figure 4 presents the Bland–Altman plots of agreement between the predicted Cai1 (a) and Cai2 (b) RMR (kcal/day) and the measured RMR for males and females. The Cai1 scatter pattern was narrow, with all but one value within the 95% LoA. Cai2 showed greater scatter and a wider lower limit for the 95% LoA than Cai1. Neither RMR prediction equation differed significantly from the measured RMR (*p* > 0.05).

### 3.10. Percent Adequacy, Overestimation, and Underestimation of the Prediction Equations

**Figure 5:**
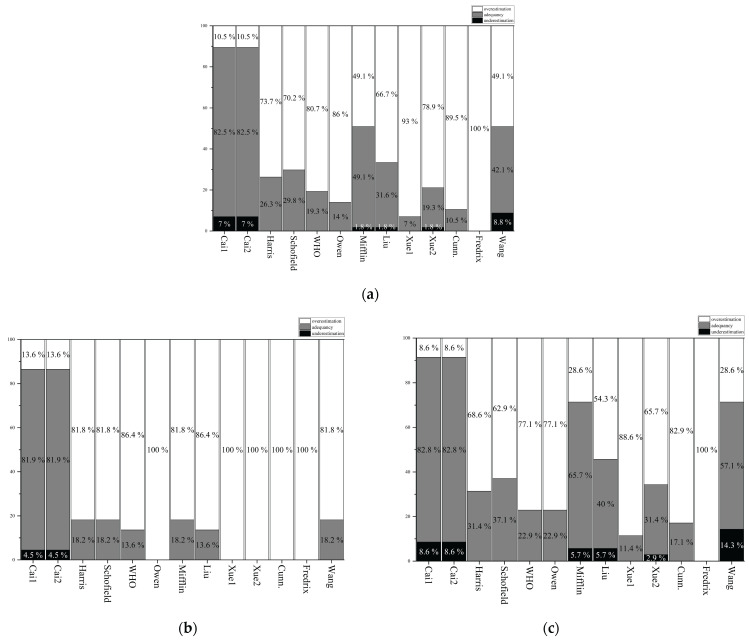
Percent adequacy, overestimation, and underestimation of predicted RMR (kcal/day) relative to measured RMR; gray color: adequacy; white color: overestimation; black color: underestimation; Cunn: Cunningham [20]. (**a**) = total sample; (**b**) = males; (**c**) = females.

Table 8 and Figure 5a present the percent adequacy, overestimation, and underestimation of the prediction equations relative to the measured RMR in the total subsample. The Cai1 and Cai2 prediction equations demonstrated excellent predictive accuracy, with percent adequacy, overestimation, and underestimation within the established limits of acceptability (±10%). In contrast, the published RMR prediction equations exhibited poor percent adequacy rates ranging from 0% to 49.1%, with overestimation ranging from 49.1% to 100% and underestimation ranging from 0% to 8.8%.

Figure 5b,c present sex-specific percent adequacy, overestimation, and underestimation values. In the male subsample, Cai1 and Cai2 both had the highest percent adequacy and lowest over- and underestimation. All 11 published prediction equations showed percent adequacy ranging from 0% to 18.2%, with overestimation ranging from 81.8% to 100%. In the female subsample, Cai1 and Cai2 showed the highest percent adequacy (82.8%) with 7.0% underestimation and 10.0% overestimation. The 11 published RMR prediction equations had poor percent adequacy rates ranging from 0% to 65.7%, with overestimation ranging from 28.6% to 100% and underestimation ranging from 2.9% to 14.3%.

## 4. Discussion

### 4.1. Key Findings

A critical gap in clinical nutritional practice for Chinese populations is the failure of published RMR prediction equations to account for the unique body composition and age-related metabolic decline of Chinese older adults (≥60 years), leading to inaccurate energy prescriptions. Collectively, most of the 11 published equations exhibited either sex-specific or total sample systematic overestimation, along with poor predictive performance. To address this gap, two novel RMR prediction equations (Cai1 and Cai2) were developed and validated for use with Chinese older adults. Cai1, which includes fat-free mass (FFM) and age as predictive variables, is an equation tailored for use in individual settings since it includes DXA-measured FFM (R^2^ = 0.572, accuracy = 82.5%); Cai2, which includes weight, age, and sex as easily measured predictive variables, is more practical for clinical and large-scale epidemiological settings since it includes predictive variables that are easily measured without sophisticated laboratory equipment (R^2^ = 0.528, accuracy = 82.5%).

In cross-validation with the measured RMR in a subsample of 57 participants, the Cai1 and Cai2 RMR prediction equations showed good agreement with the measured RMR, negligible systematic bias (0.72% to 1.08%), low $\bar{x}$_ds (Cai1 = +8.0 kcal, Cai2 = +12.0 kcal, *p* = 1.000), and narrower limits of agreement (LoA: Cai1 = −170.4 kcal to +154.3 kcal and Cai2 = −175.7 kcal to +151.6 kcal). In addition, the percent adequacy of both equations was 82.5%, with values ranging from 90% to 110% of the measured RMR. In contrast, the 11 widely used published prediction equations consistently overestimated measured RMR with $\bar{x}$_ds ranging from +97.1 kcal to +520.8 kcal, LoA exceeding ~ +300 kcal to +800 kcal, and systematic biases ranging from +8.39% to +38.03%. Also, the percent adequacy of the published equations was poor, ranging from 0% to 49.1%. Stratification by age (<80 and ≥80 years) showed the prediction equations performed poorly in adults aged ≥80, limiting their use to those aged <80. Collectively, the favorable metrics for the Cai1 and Cai2 RMR prediction equations indicate a clear advantage for estimating RMR in Chinese older adults aged <80.

### 4.2. Underlying Mechanism for Discrepancies in Published RMR Prediction Equations

#### 4.2.1. Body Composition Drives Bias

The fundamental limitation of the 11 published RMR prediction equations lies in their derivation from White Caucasian and non-geriatric populations—populations that differ markedly in body composition from Chinese older adults. FFM is the primary factor affecting RMR. Collectively, the FFM and measured RMR in this sample were 41.5 ± 7.9 kg and 1116.9 ± 147.9, respectively. The males had a mean FFM of 49.3 ± 5.6 kg and an RMR of 1197.0 ± 152.9 kcal, whereas the females had a lower FFM and RMR (36.6 ± 4.5 kg; 1071.2 ± 158.9 kcal, respectively) than the males (see Table 2). The FFM and RMR values for participants in this sample are significantly lower than those reported in Western older adults (age range: 60–91; male FFM = 57.8 ± 9.1 kg, RMR = 1653.0 ± 234.0 kcal; female FFM = 42.3 ± 7.8 kg, RMR = 1340.0 ± 155.0 kcal; total sample FFM = 48.7 ± 9.5 kg, RMR = 1436.0 ± 232.0 kcal) [36]. The larger difference in FFM in Western older adults is sizable compared to Chinese older adults (male: +8.5 kg vs. female: +5.7 kg), which in turn leads to a greater degree of overestimation when these prediction equations were applied to predict RMR in the current sample (males: 81.8%~100% in overestimation, with five equations reaching 100% overestimation [17,20,21,24]; females: 28.6%~100% in overestimation, with one equation reaching 100% overestimation [21]). Further, Chinese and Western populations differ in average body weight, with Western older adults [36] weighing ~8.2 kg more than Chinese older adults in this study (71.8 kg vs. 63.6 kg). These differences in FFM and body weight highlight inherent demographic and ethnic differences that the published prediction equations fail to account for by omitting FFM (n = 7 [14,15,16,17,18,21]) and body weight (n = 3 [Cunningham [20], Wang [23], Xue2 [24]]). This discrepancy in body composition contributes to the predictive inaccuracy of RMR prediction equations developed in Western populations when applied to the current study sample.

Highlighting the importance of FFM in predicting RMR, FFM emerged as the dominant predictor of RMR in novel equation Cai1 (β_FFM_ = 0.632), with a substantially higher regression coefficient than the predictive variable age (β_AGE_ = −0.445; Table 3). Including FFM in Cai1 resulted in a slightly higher coefficient of determination (R^2^ = 0.572) than in Cai2 (R^2^ = 0.528), which did not include FFM. Interestingly, none of the RMR prediction equations included fat mass as a predictive variable, including Cai1 and Cai2. Fat mass and fat distribution play a negligible role in determining RMR, whereas FFM is the most important predictive variable of RMR [37].

Pearson correlation coefficients between the predicted and measured RMR for the novel (r = 0.80–0.83) and published RMR prediction equations (r = 0.73–0.87) were rated as moderately to very strong (r > 0.6) in males. In females, correlations for the novel equations (r = 0.65–0.68) were rated as moderately strong, and the published equations (r = 0.19–0.51) were rated as poor to fairly strong. This discrepancy may stem from sex-specific body composition dynamics, hormonal fluctuations, and differences in fat distribution. In this study, males had more fat-free mass, ranging from 41.7 to 65.1 kg (a 23.4 kg difference), higher body weight, ranging from 53.0 to 87.6 kg (a 34.6 kg difference), and less fat mass, ranging from 9.2 to 32.1 kg (a 22.1 kg difference) compared with females. Alternatively, all females were postmenopausal, which promotes estrogen decline and is associated with the acceleration of sarcopenia and central obesity. Accordingly, females had a lower fat-free mass, ranging from 28.8 to 46.5 kg (a 17.7 kg difference), a wider range in body weight from 37.0 to 88.9 kg (a 51.9 kg difference), and a greater fat mass, ranging from 8.2 to 42.4 kg (a 34.2 kg difference) compared with males. Predicted RMR is heavily influenced by fat-free mass, which may explain the higher correlations in males than in females. Lower fat-free mass and greater variation in body weight and fat mass in females amplify the heterogeneity of metabolically active tissues, weakening the linear correlation between predictor variables and measured RMR. Notably, three widely used published prediction equations (Schofield [16], WHO [15], and Owen [17]) that excluded FFM and age showed extremely weak correlations with measured RMR in each equation, r = 0.19 (Table 6), whereas Cai1, which included FFM as a predictive variable, showed a correlation of r = 0.68. Although weak linear correlations were observed in the female subsample, correlations in the total validation sample were only fair to moderately strong. This range of correlation coefficients underscores the importance of using RMR prediction equations that are appropriate for a population’s demographic and anthropometric characteristics.

The published prediction equations also yielded significant overestimation in predicted RMRs. For example, the Owen [17] equation overestimated RMR by 21.54% (Table 8) and achieved only 14% accuracy in the validation total sample. In contrast, Cai1 and Cai2 maintained a high percent accuracy (82.5%). This level of percent accuracy indicates higher precision in predicting RMR among Chinese older adults than with published prediction equations.

#### 4.2.2. Unexpected Results from RMR Prediction Equations Derived from Chinese Populations

Among the 11 validated RMR prediction equations, four were developed in Chinese populations (Liu [22], Wang [23], Xue1 [24], and Xue2 [24]). A surprising finding was that these Chinese-specific equations failed to provide acceptable predictive accuracy. Xue1 had the largest overestimation (systematic bias = 28.56%, $\bar{x}$_d = 369.4 ± 169.6 kcal, 95% CI for bias of +324.4 to +414.4 kcal) with only 7% adequacy and 93% overestimation in participants in the validation sample. In the male subsample, the Xue1 prediction equation had 0% adequacy, 100% overestimation, and a systematic bias of 44.30%. Even though incorporating FFM as a strong predictive variable increased the predictive adequacy of the Xue2 [24] equation (from 7.0% to 19.2%), systematic bias remained (20.36%) in males (31.22%) and in females (16.76%). The other RMR prediction equations developed in Chinese populations showed consistent systematic bias (ranging from 8.39% to 28.56%), with higher biases in males (17.20% to 44.30%) than in females (2.93% to 25.73%). These results suggest that additional factors are important for predicting RMR beyond ethnicity.

#### 4.2.3. Unexpected Results from RMR Prediction Equations Derived from Different Age Samples

The age of the sample is a critical component in identifying an RMR prediction equation for use in specific populations. The systematic bias in the Chinese population RMR prediction equations may have been due to the younger age range of participants in the published studies than in the current study. For example, the three prediction equations developed in Chinese populations had age ranges that differed from the current study’s 60–94 years (Xue1 and 2 [24] = 18–67; Wang [23] ≤ 45; Liu [22] = 20–78). Surprisingly, Wang’s [23] equation was the most accurate, with the smallest systematic bias and the highest percent adequacy among the four Chinese-specific equations. Nevertheless, it still showed a systematic bias of 8.39% and an adequacy of only 42.1%. This imprecision may be due to its failure to include age as a predictive variable in the RMR prediction equation. However, it is unlikely that age is a primary factor in the failure of published equations to accurately predict RMR in the current sample. Two of 11 published prediction equations were developed for older adults (Schofield [16], aged ≥60; Fredrix [21], aged 51–82). Still, both equations overpredicted RMR by 15.38% to 38.03% in the current sample. Further, neither equation included FFM as a predictive variable, which may have contributed to their inaccuracy in predicting RMR in the current sample.

Aging induces progressive physiological changes that reduce RMR independently of body composition, and these declines often co-occur with reductions in FFM, bone density, organ mass [9,38], decreases in physical activity, and an increased risk of chronic diseases. This study highlighted age’s critical role in predicting RMR, as age was a strong negative predictor in both novel equations (Cai1: β_age_ = −0.445; Cai2: β_age_ = −0.461). Significantly, reductions in RMR are associated with metabolic decline and other physiological processes associated with aging. Studies have shown that when adults reach very old age (e.g., ≥90), adjusted TEE is ~26% lower than that of middle-aged adults (~40 to 70 years), even after accounting for changes in body composition, namely FFM [10,39]. This change is partly driven by reduced metabolic activity in highly metabolically active organs (e.g., brain, liver, heart, and kidney), which contribute 60 to 70% of RMR despite comprising <6% of body weight [40]. Additional mechanisms—such as decreased Na^+^/K^+^-ATPase activity, impaired mitochondrial function, and reduced growth hormone levels—further exacerbate RMR’s decline in aging [38]. Alternatively, maintenance of FFM is associated with higher RMR values [36], underscoring the importance of engaging in regular physical activity and resistance training to maintain FFM in older adults.

Aside from physiological and metabolic changes that occur with aging, the recruitment of study participants is difficult among very old adults (i.e., aged ≥80). Only 5% of the participants in the current study were aged ≥80. Compared with participants aged <80, the regression slope and intercept values were wider and more unstable for predicting RMR than for participants aged <80. Based on the small sample of participants aged ≥80 (n = 6 in validation sample, n = 10 in total sample) and the unstable regression coefficients, the Cai1 and Cai2 RMR prediction equations are limited to adults aged <80.

### 4.3. Potential Applications of the Novel Equations

Erroneous RMR overestimation in practice may lead to excessive energy supply, exacerbating obesity-related comorbidities and sarcopenic obesity of Chinese older adults [4]. Given that 34.8% of Chinese older adults are overweight and 12.4% are obese, and this is more prevalent among those living in urban areas (overweight = 40.0% and obesity = 14.8%) than in rural areas (overweight = 28.2% and obesity = 9.3%) [4], the RMR prediction equations generated from this study support the primary prevention of both undernutrition and overnutrition, aligning with global geriatric nutrition guidelines that emphasize ethnicity-specific metabolic assessment [2].

The population-specific precision of the novel Cai1 and Cai2 RMR prediction equations confers substantial translational value for Chinese older adults, with three key stakeholder groups poised to leverage these findings. Clinical dietitians can utilize the Cai1 equation (FFM-based) to optimize energy prescriptions for older adults. In contrast, the Cai2 equation (weight-based) is suitable for individuals without access to DXA. Public health researchers and policymakers can utilize the RMR prediction equations to accurately stratify population-level nutritional risks and inform evidence-based policy design for community meal programs and sarcopenia prevention campaigns, thereby reducing estimation error in large-scale surveys and epidemiological studies. Exercise physiologists and geriatric fitness coaches benefit from having precise RMR estimates that help calibrate safe, individualized exercise intensity and duration, as well as chronic disease-adapted physical activity prescriptions to avoid overexertion or metabolic stress.

### 4.4. Strength and Limitations

A key strength of our study is the rigorous control of potential confounding factors, including multimorbidity [30], the exclusion of medical conditions known to affect RMR [26] (e.g., uncontrolled diabetes or thyroid disorders), and the mitigation of thermogenic effects from food, caffeine, nicotine, and physical activity before RMR measurement [18]. Additionally, gold-standard methods were used for assessing both RMR (IC) and body composition (DXA) [41], techniques widely recognized as reference standards in metabolic research. Secondly, the study design included a development subsample (n = 132) and a validation subsample (n = 57), with stepwise regression to select predictive variables and strict control for multicollinearity (VIF < 5) and autocorrelation (D–W = 1.631), ensuring the stability and reliability of the Cai1 and Cai2 RMR prediction equations. Thirdly, the study employed a comprehensive validation framework, including paired *t*-test and RM-ANOVA to assess $\bar{x}$_ds, Bland–Altman analysis to evaluate agreement and systematic bias, and Pearson product–moment correlations to measure the strength of linear associations. Multi-stage analysis captured clinical agreement; for example, the Fredrix [21] equation showed a strong correlation in males (r = 0.83), but Bland–Altman analysis and percent adequacy evaluation showed a $\bar{x}$_d of +377.5 kcal and a LoA of +526.1 to +228.8 kcal, providing strong evidence of failure for clinical use. Lastly, a total of 11 published RMR prediction equations were cross-validated in this study, including widely used equations [14,15,16,17,18] developed specifically for older adults [21], and equations developed for use in Chinese populations [22,23,24]. The published equations evaluated in this study encompass all applicable prediction equations for RMR assessment in Mainland Chinese older adults.

Nevertheless, several limitations of the present study need to be addressed. Firstly, the validation cohort was relatively small, consisting of only 57 participants. Stratifying the validation sample by sex resulted in even smaller subsamples (males = 22; females = 35). As presented in the discussion, the lower levels of FFM in females than males contributed to the lower correlations and R^2^ values between the predicted and measured RMR values in females than in males. Secondly, despite the broad age distribution of the study participants (60–94 years), the majority (95%) were aged 60–79, with an average age of 69.5 ± 6.3. Recruiting participants aged ≥80 was difficult at community centers in Shanghai, as few older adults attending were eligible for the study due to its inclusion criteria and life expectancy in Shanghai. Nearly 95% of adults aged ≥80 have multimorbidity [42], and, in 2023, life expectancy in Shanghai was 80.84 years for males and 85.66 years for females [43]. Further, results from ICC estimation and bootstrap validation demonstrated the novel prediction equations were unstable and insensitive to sampling variation, hence the Cai1 and Cai2 prediction equations should not be applied to older adults aged ≥80. Future research should prioritize investigating RMR in older adults with and without multimorbid conditions to expand population diversity in adults, with the majority of participants aged ≥80 and to optimize the Cai1 and Cai2 prediction equations for broader clinical utility.

Further, although participants were raised in diverse regions of China, all resided in Shanghai at the time of data collection. This distribution may limit generalizability, as regional differences in current lifestyle (e.g., physical activity levels), dietary patterns (e.g., protein intake) [3], and body composition (e.g., regional variations in muscle mass) can influence RMR. Therefore, multicenter studies across multiple Chinese provinces are needed to improve the applicability of the Cai1 and Cai2 prediction equations. While rigorous control of participants’ health status ensured strong predictive ability, older populations in clinical and community settings frequently have comorbidities (e.g., chronic heart failure, or chronic obstructive pulmonary disease) that modulate RMR [44]. To gain a better understanding of the RMR in healthy older adults living in Shanghai, the participants in this study were relatively healthy and absent from complex comorbidities. Therefore, the application of current equations should be cautious in people with multimorbidity and to other regions in China, especially those individuals with chronic disease that alter RMR and older adults taking medications that modify RMR.

## 5. Conclusions

Studies have demonstrated that a single RMR prediction equation cannot be accurately applied to populations that differ in age, sex, ethnicity, or physical status from the population for which it was developed [45]. Published RMR prediction equations are unsuitable for Chinese older adults because they fail to account for ethnic-specific body composition, accelerated age-related metabolic decline, and inadequate selection of predictive variables. The novel equations developed in this study incorporate FFM and age (Cai1), body weight, age and sex (Cai2), providing a clinically valid tool for predicting RMR and individualized energy intake prescriptions for older adults aged <80. The results of this study reinforce the need for population-specific RMR prediction equations to optimize nutrition care in aging populations and lay the groundwork for future studies integrating biological markers to further refine RMR predictions. Future studies should validate the RMR prediction equations developed in this study in adults aged ≥80, and in multicenter settings, including rural and northern Chinese older adults; incorporate comorbidities and biological markers to improve accuracy in clinical populations; and explore the equations’ utility in sarcopenia prevention programs.

## Figures and Tables

**Table 1:** Published Resting Metabolic Rate (RMR, kcal/day) prediction equations.

Equation	Characteristics	Population	Equation
Harris–Benedict (1918) [14]	N = 239 (136 M, 103 F)	Male Female	RMR = 66 + (13.7 × WT) + (5 × HT) − (6.8 × age) RMR = 655 + (9.5 × WT) + (1.9 × HT) − (4.7 × age)
	Age = 29 ± 14		
Schofield (1985) [16]	N = 86 (50 M, 36 F)	Male Female	RMR = (11.711 × WT) + 587.7 RMR = (9.082 × WT) + 658.5
	Age > 60		
WHO (1985) [15]	N ≈ 2526 (2279 M, 247 F)	Male Female	RMR = (13.5 × WT) + 487.0 RMR = (10.5 × WT) + 596.0
	Age = 19–82		
Owen (1986) [17]	N = 104 (60 M, 44 F)	Male Female	RMR = (10.2 × WT) + 879.0 RMR = (7.18 × WT) + 795.0
	Age = 18–82		
Mifflin–St. Jeor (1990) [18]	N = 498 (251 M, 248 F)	Male Female	RMR = (10 × WT) + (6.25 × HT) − (5 × age) + 5.0 RMR = (10 × WT) + (6.25 × HT) − (5 × age) − 161
	Age = 19–78		
Liu (1995) [22]	N = 223 (102 M, 121 F)	All	RMR = (13.88 × WT) + (4.16 × HT) − (3.43 × age) − (112.4 × sex)
	Age = 20–78		
Xue equation 1 (2019) [24] (Xue1)	N = 315 (127 M, 188 F)	All	RMR = (13.9 × WT) + (247 × sex) − (5.39 × age) + 855.0
	Age = 18–67		
Cunningham (1980) [20]	N = 223 (136 M, 103 F)	All	RMR = (21.6 × FFM) + 501.6
	Age = 29 ± 11		
Fredrix (1990) [21]	N = 40 (18 M, 22 F)	All	RMR = (10.7 × WT) − (9 × age) − (203 × sex) + 1641.0
	Age = 65 ± 8		
Wang (2000) [23]	N = 174	All	RMR = (24.6 × FFM) + 175.0
	Age < 45		
Xue equation 2 (2019) [24] (Xue2)	N = 315 (127 M, 188 F)	All	RMR = (26.535 × FFM) − (5.06 × age) + 602.1
	Age = 18–67		

M = male; F = female; Age is in years; WT = body weight in kg; FFM = fat-free mass in kg; sex in Fredrix: M = 1, F = 2; sex in Liu: M = 0, F = 1; sex in Xue1: M = 1, F = 0.

**Table 2:** Mean ± SD and range for descriptive, anthropometric, and cardiovascular data of study participants in the total sample (N = 189) and by sex (males = 73; females = 116).

Variable	Total (N = 189)	Males (n = 73)	Females (n = 116)
	Mean ± SD	Mean ± SD	Mean ± SD
	(Range)	(Range)	(Range)
Measured RMR (Kcal/Day)	1116.9 ± 147.9	1197.0 ± 152.9	1071.2 ± 158.9
	(689–1530)	(863–1530)	(689–1470)
FFM (kg)	41.5 ± 7.9	49.3 ± 5.6	36.6 ± 4.5
	(28.8–65.1)	(41.7–65.1)	(28.8–46.5)
Age (y)	69.5 ± 6.3	70.8 ± 6.1	68.6 ± 6.2
	(60–94)	(60–94)	(60–92)
Weight (kg)	63.6 ± 10.5	70.2 ± 8.9	59.5 ± 9.3
	(37.0–88.9)	(53.0–87.6)	(37.0–88.9)
Height (cm)	162.7 ± 7.5	169.7 ± 5.2	158.2 ± 4.9
	(143.0–186.7)	(158.0–186.7)	(143.0–168.3)
BMI (kg/m^2^)	24.0 ± 3.1	24.3 ± 2.7	23.7 ± 3.4
	(15.8–35.5)	(20.3–30.6)	(15.8–35.5)
FM (kg)	22.1 ± 5.8	20.9 ± 5.0	22.9 ± 6.1
	(8.2–42.4)	(9.2–32.1)	(8.2–42.4)
FM (%)	34.7 ± 6.6	29.5 ± 4.5	38.0 ± 5.5
	(16.9–47.7)	(16.9–36.9)	(19.6–47.7)
SBP (mmHg)	128.7 ± 13.1	132.6 ± 13.1	126.5 ± 12.5
	(93–150)	(93–147)	(97–150)
DBP (mmHg)	78.8 ± 8.8	81.9 ± 8.4	77.1 ± 8.6
	(61–94)	(68–94)	(61–94)

SD: standard deviation; BMI: body mass index; FM: fat mass; FFM: fat-free mass; measured RMR: resting metabolic rate measured by indirect calorimetry; SBP: systolic blood pressure; DBP: diastolic blood pressure.

**Table 3:** Cai1 and Cai2 RMR (kcal/day) prediction equations based on fat-free mass, age, sex, and weight in the development subsample (n = 132).

Equation	Regression Equation	*p*	R^2^	β	RMSE
Cai1	RMR = 1393.019 − (11.112 × age) + (11.963 × FFM)	<0.01	0.572	age (−0.445) FFM (0.632)	99.238
Cai2	RMR = 1537.513 + (91.038 × sex) − (11.515 × age) + (5.436 × WT)	<0.01	0.528	age (−0.461) sex (0.292) WT (0.387)	104.189

R^2^: coefficient of determination; RMSE = root mean squared error; RMR: resting metabolic rate; FFM: fat-free mass in kg; WT: weight in kg; sex: male = 1, female = 0.

**Table 4:** Regression analysis of factors associated with RMR (n = 132).

Equation	Variable	β (95% CI)	SE	*p*
Cai1 equation	Constant	1393.019 (1179.673, 1606.365)	107.831	<0.001
	Age	−11.112 (−13.961, −8.264)	1.440	<0.001
	FFM	11.963 (9.803, 14.123)	1.092	<0.001
Cai2 equation	Constant	1537.513 (1273.273, 1801.753)	133.544	<0.001
	Age	−11.515 (−14.638, −8.388)	1.581	<0.001
	Sex	91.038 (44.862, 137.213)	23.337	<0.001
	Weight	5.436 (3.401, 7.471)	1.028	<0.001

Sex: Male = 1, Female = 0; β (95% CI) = slope and 95% confidence interval; SE = standard error.

**Table 5:** $\bar{x}$_d between the RMR (kcal/day) prediction equations and the measured RMR for the total validation subsample (n = 57) and by sex (males, n = 22; females, n = 35).

Variable	Predicted RMR (kcal/day)	Measured RMR (kcal/day)	$\bar{x}$_d (kcal/day)	t	*p*
Cai1	1117.0	1109.0	8.0	0.53	1.000 ^a^
Male	1193.7	1175.3	18.4	1.25	0.993 ^a^
Female	1068.8	1067.3	1.5	0.12	1.000 ^a^
Cai2	1121.0	1109.0	12.0	0.80	1.000 ^a^
Male	1197.1	1175.3	21.8	1.47	0.971 ^a^
Female	1073.2	1067.3	5.9	0.46	1.000 ^a^
Harris [14]	1285.8	1109.0	176.8	11.69	<0.001
Male	1392.7	1175.3	217.4	14.70	<0.001
Female	1218.6	1067.3	151.3	11.67	<0.001
Schofield [16]	1293.7	1109.0	184.7	12.21	<0.001
Male	1408.2	1175.3	232.9	15.75	<0.001
Female	1221.7	1067.3	154.5	11.92	<0.001
WHO [15]	1318.9	1109.0	209.9	13.87	<0.001
Male	1432.8	1175.3	257.5	17.42	<0.001
Female	1247.2	1067.3	179.9	13.88	<0.001
Owen [17]	1376.7	1109.0	267.7	17.69	<0.001
Male	1593.6	1175.3	418.3	28.29	<0.001
Female	1240.3	1067.3	173.0	13.35	<0.001
Mifflin [18]	1222.0	1109.0	113.0	7.47	<0.001
Male	1411.1	1175.3	235.8	15.95	<0.001
Female	1103.0	1067.3	35.8	2.76	0.249 ^a^
Liu [22]	1272.2	1109.0	163.2	10.78	<0.001
Male	1434.8	1175.3	259.5	17.55	<0.001
Female	1169.8	1067.3	102.6	7.92	<0.001
Xue1 [24]	1478.4	1109.0	369.4	24.42	<0.001
Male	1695.6	1175.3	520.3	35.19	<0.001
Female	1341.9	1067.3	274.6	21.19	<0.001
Xue2 [24]	1360.3	1109.0	251.3	16.61	<0.001
Male	1541.9	1175.3	366.6	24.79	<0.001
Female	1246.1	1067.3	178.9	13.80	<0.001
Cunningham [20]	1407.0	1109.0	298.0	19.70	<0.001
Male	1557.2	1175.3	381.9	25.83	<0.001
Female	1312.5	1067.3	245.2	18.93	<0.001
Fredrix [21]	1629.8	1109.0	520.8	34.42	<0.001
Male	1552.7	1175.3	377.5	25.53	<0.001
Female	1678.2	1067.3	610.9	47.14	<0.001
Wang [23]	1206.1	1109.0	97.1	6.42	<0.001
Male	1377.2	1175.3	201.9	13.66	<0.001
Female	1098.6	1067.3	31.3	2.42	0.470 ^a^

^a^: no statistically significant difference; $\bar{x}$_ds calculated by predicted RMR kcal-measured RMR kcal; Harris: Harris–Benedict [14]; Mifflin: Mifflin–St. Jeor [18].

**Table 6:** Pearson product–moment correlations between the RMR (kcal/day) prediction equations and the measured RMR (kcal/day) for the total validation subsample (n = 57) and by sex (males = 22; females = 35).

Equations	Mean ± SD (kcal/day)			Pearson’s Correlation Coefficient (r)		
	Male (n = 22)	Female (n = 35)	Total (n = 57)	Male	Female	Total
Measured RMR	1173.3 ± 125.6	1067.3 ± 112.0	1108.9 ± 128.7			
Cai1	1193.7 ± 121.4	1068.8 ± 102.0	1117.0 ± 125.6	0.83 ^a^	0.68 ^a^	0.79 ^a^
Cai2	1197.1 ± 104.0	1073.2 ± 89.2	1121.0 ± 112.7	0.80 ^a^	0.65 ^a^	0.77 ^a^
Harris [14]	1392.7 ± 161.5	1218.6 ± 93.2	1285.8 ± 150.3	0.78 ^a^	0.43 ^b^	0.69 ^a^
Schofield [16]	1408.2 ± 107.3	1221.7 ± 79.6	1293.7 ± 128.7	0.73 ^a^	0.19	0.58 ^a^
WHO [15]	1432.8 ± 123.7	1247.2 ± 92.0	1318.8 ± 138.8	0.73 ^a^	0.19	0.58 ^a^
Owen [17]	1593.6 ± 93.5	1240.3 ± 62.9	1376.6 ± 188.1	0.73 ^a^	0.19	0.54 ^a^
Mifflin [18]	1411.1 ± 127.0	1103.0 ± 110.6	1221.9 ± 190.4	0.75 ^a^	0.45 ^a^	0.65 ^a^
Liu [22]	1434.8 ± 149.2	1169.8 ± 133.4	1272.1 ± 190.2	0.75 ^a^	0.35 ^b^	0.63 ^a^
Xue1 [24]	1695.6 ± 142.3	1341.9 ± 126.6	1478.4 ± 217.5	0.80 ^a^	0.37 ^b^	0.64 ^a^
Xue2 [24]	1541.9 ± 163.2	1246.1 ± 139.9	1360.3 ± 207.4	0.87 ^a^	0.50 ^a^	0.72 ^a^
Cunn. [20]	1557.2 ± 116.5	1312.5 ± 105.5	1407.0 ± 162.0	0.85 ^a^	0.37 ^b^	0.66 ^a^
Fredrix [21]	1552.7 ± 129.6	1678.2 ± 110.5	1629.7 ± 133.1	0.83 ^a^	0.51 ^a^	0.35 ^a^
Wang [23]	1377.2 ± 132.7	1098.6 ± 120.2	1206.1 ± 184.6	0.85 ^a^	0.37 ^b^	0.66 ^a^

^a^ *p* < 0.05; ^b^ *p* < 0.01; Harris: Harris–Benedict [14]; Mifflin: Mifflin–St. Jeor [18]; Cunn: Cunningham [20].

**Table 7:** $\bar{x}$_d and standard deviation, limits of agreement, 95% confidence interval of the $\bar{x}$_d, and *p*-values of the predicted and measured RMR (kcal/day) in the total validation subsample (n = 57) and by sex (males, n = 22; females, n = 35).

RMR Prediction Equation	$\bar{x}$_d ± SD ^a^ (kcal/day)			LoA ^a^ ($\bar{x}$_d ± 1.96 SD) (kcal/day)			95% CI ^a^ of the $\bar{x}$_d (kcal/day)			*p*
	Male (n = 22)	Female (n = 35)	Total (n = 57)	Male (n = 22)	Female (n = 35)	Total (n = 57)	Male (n = 22)	Female (n = 35)	Total (n = 57)	Total (n = 57)
Cai1	18.5 ± 74.9	1.5 ± 87.9	8.0 ± 82.8	−128.4, 165.3	−170.7, 173.7	−154.3, 170.4	−14.8, 51.7	−28.7, 31.7	−13.9, 30.0	0.466
Cai2	21.8 ± 77.4	5.9 ± 87.7	12.0 ± 83.5	−129.9, 173.5	−165.9, 177.8	−151.6, 175.7	−12.5, 56.1	−24.2, 36.0	−10.1, 34.2	0.281
Harris [14]	217.4 ± 103.9	151.3 ± 112.7	176.8 ± 113.2	13.8, 421.0	−69.5, 372.1	−45.0, 398.6,	171.4, 263.5	112.6, 190.0	146.8, 206.8	<0.05
Schofield [16]	232.9 ± 89.9	154.5 ± 126.0	184.7 ± 119.0	56.6, 409.2	−92.5, 401.5	−48.5, 418.0	193.0, 272.8	111.2, 197.7	153.1, 216.3	<0.05
WHO [15]	257.5 ± 94.5	179.9 ± 132.4	209.9 ± 124.2	72.3, 442.7	−79.5, 439.3	−33.7, 453.4	215.6, 299.4	134.4, 225.4	176.9, 242.8	<0.05
Owen [17]	418.3 ± 88.5	173.0 ± 119.1	267.7 ± 161.4	245.0, 591.7	−60.4, 406.4	−48,7, 584.1	379.1, 457.6	132.1, 213.9	224.9, 310.5	<0.05
Mifflin [18]	235.8 ± 91.3	35.8 ± 118.1	113.0 ± 145.7	57.0, 414.7	−195.6, 267.2	−172.7, 398.6	195.4, 276.3	−4.8, 76.3	74.3, 151.7,	<0.05
Liu [22]	259.5 ± 101.9	102.6 ± 143.6	163.2 ± 149.5	59.8, 459.3	−178.8, 383.9	−129.9, 456.2	214.3, 304.7	53.3, 151.9	123.5, 202.8,	<0.05
Xue 1 [24]	520.3 ± 87.4	274.6 ± 136.7	369.4 ± 169.6	349.0, 691.7	6.7, 542.5	36.9, 701.9	481.5, 559.1	227.6, 321.6	324.4, 414.4	<0.05
Xue 2 [24]	366.6 ± 83.7	178.9 ± 130.3	251.3 ± 146.4	202.6, 530.6	−76.5, 434.3	−35.6, 538.3	329.5, 403.7	134.1, 223.6	212.4, 290.2	<0.05
Cunningham [20]	381.9 ± 68.7	245.3 ± 123.8	298.0 ± 124.8	247.3, 516.4	2.6, 488.0	53.4, 542.6	351.4, 412.3	202.8, 287.8	264.9, 331.1	<0.05
Fredrix [21]	377.5 ± 75.8	610.9 ± 111.7	520.8 ± 151.2	228.8, 526.1	392.0, 829.8	224.3, 817.2	343.8, 411.1	572.5, 649.2	480.7, 560.9	<0.05
Wang [23]	201.9 ± 73.0	31.3 ± 132.2	97.1 ± 140.1	58.9, 344.9	−227.8, 290.4	−177.4, 371.7	169.5, 234.2	−14.1, 76.7	60.0, 134.3	<0.05

^a^ $\bar{x}$_d calculated by Predicted RMR-measured RMR (kcal/day) ($\bar{x}$_d ± SD); LOA = limits of agreement; CI = Confidence Interval; Harris: Harris–Benedict [14]; Mifflin: Mifflin–St. Jeor [18].

**Table 8:** Percent adequacy, overestimation, and underestimation of predicted RMR kcal/day relative to measured RMR (kcal/day) in the validation subsample (n = 57).

Prediction Equation	Adequacy %	Overestimation %	Underestimation %
Cai1	82.5	10.5	7.0
Cai2	82.5	10.5	7.0
Harris–Benedict [14]	26.3	73.7	0
Schofield [16]	29.8	70.2	0
WHO [15]	19.3	80.7	0
Owen [17]	14	86	0
Mifflin [18]	49.1	49.1	1.8
Liu [22]	31.6	66.7	1.8
Xue1 [24]	7	93	0
Xue2 [24]	19.3	78.9	1.8
Cunningham [20]	10.5	89.5	0
Fredrix [21]	0	100	0
Wang [23]	42.1	49.1	8.8

Note: Values for predicted kcal vs. measured kcal are underestimation <10%; overestimation >10%; adequacy 90–110%. Mifflin: Mifflin–St. Jeor [18].

## Data Availability

The data presented in this study are available on request from the corresponding author due to ethical restrictions.

## Supplementary Materials

The following supporting information can be downloaded at: https://www.mdpi.com/article/10.3390/nu18020344/s1, Table S1: 10-fold cross-validation analysis to assess the certainty of the validation sample size; Table S2: Intraclass correlation analysis to determine absolute agreement between the Cai1 and Cai2 RMR (kcal/day) prediction equations and measured RMR (kcal/day); Table S3: Prespecified age-stratified sensitivity analysis with internal bootstrap validation using 1000 resamples in participants aged <80 and aged ≥ 80 years for Cai1 and Cai2 RMR prediction equations.
