# Supplementary material for: Prediction Equations to Estimate Resting Metabolic Rate in Healthy, Community-Dwelling Chinese Older Adults
Source: Nutrients. 2026 Jan 21;18(2):344. doi: 10.3390/nu18020344 (PMC12844918; doi:10.3390/nu18020344)
Supplement: Supplementary file 1 [file nutrients-18-00344-s001.zip › nutrients-4090533-supplementary.pdf]

Supplementary Tables

Supplementary Table S1. 10-fold cross-validation analysis to assess the certainty of the validation sample size.

	Equation	R^2（10-fold）	RMSE (10-fold)
Cai1 (n=132)	RMR = 1393.019 – (11.112 x age) + (11.963 x FFM)	0.572	99.238
Cai1 cross-validation (N=189)	RMR = 1351.8 – (10.17 x age) + (11.324 x FFM)	0.574（0.565）	92.920 (95.704)
Cai2 (n=132)	RMR = 1537.513 + (91.038 x sex) - (11.515 x age) + ((5.436 x WT)	0.528	104.189
Cai2 cross-validation (N=189)	RMR = 1524 + (88.1 x sex) - (11.14 x age) + ((5.192 x WT)	0.523 (0.521)	99.349 (100.415)

Supplementary Table S2. Intraclass correlation analysis to determine absolute agreement between the Cai1 and Cai2 RMR (kcal/day) prediction equations and measured RMR (kcal/day).

	Measurement	ICC	95% CI
Cai1	Single measurement (A,1)	0.793	0.672 - 0.873
	Average measurement (A, K)	0.884	0.804 - 0.932
Cai2	Single measurement (A,1)	0.765	0.632 - 0.855
	Average measurement (A, K)	0.867	0.774 - 0.922

Note: ICC = intraclass correlation; CI = confidence interval

Supplementary Table S3. Prespecified age-stratified sensitivity analysis with internal bootstrap validation using 1000 resamples in participants aged <80 and aged ≥ 80 years for Cai1 and Cai2 RMR prediction equations.

	**Age group**	**Slope (95% CI)**	**Intercept (95% CI)**	**R^2**
Cai1	< 80	1.021 (0.836-1.197)	-25.603 (-225.377–191.682)	0.556
	≥80	1.541 (1.355–1.748)	-382.769 (-541.525–-194.784)	0.995
Cai2	< 80	1.009 (0.834-1.184)	-11.791 (-208.829-185.248)	0.507
	≥80	1.654 (0.784-2.524)	-513.158 (-663.496—437.251)	0.998
